# Prognostic Factors of Primary Intraosseous Squamous Cell Carcinoma (PIOSCC): A Retrospective Review

**DOI:** 10.1371/journal.pone.0153646

**Published:** 2016-04-13

**Authors:** Xu Wenguang, Shen Hao, Qi Xiaofeng, Wang Zhiyong, Wang Yufeng, Hu Qingang, Han Wei

**Affiliations:** 1 Department of Oral and Maxillofacial Surgery, Nanjing Stomatological Hospital, Medical School of Nanjing University, Nanjing, P.R China; 2 Central Laboratory of Stomatology, Nanjing Stomatological Hospital, Medical School of Nanjing University, Nanjing, P.R China; Kaohsiung Chang Gung Memorial Hospital, TAIWAN

## Abstract

**Objectives:**

To delineate clinical and pathological features and determine the prognostic factors of primary intraosseous squamous cell carcinoma (PIOSCC).

**Materials and methods:**

Patients diagnosed with PIOSCC, attending the department of oral and maxillofacial surgery, Nanjing stomatological hospital between 2005 and 2015, were identified and retrospectively reviewed for clinical and pathological characteristics. Therapeutic modalities were measured and related follow-up data recorded, in order to determine prognostic factors of PIOSSC.

**Results:**

A total of 77 patients with PIOSCC were included in the study. Mean age at diagnosis was 58.8 years, (range, 37−81 years). Of the 77 patients, there were 58 men and 19 women. The most common location of disease was the mandible (71.42%), particularly the posterior mandible. The common presenting symptoms included jaw swelling (79.2%) and ulceration (42.65%). The estimated 2-year and 5-year overall survival were 68.9% and 38.8%, respectively. Univariate analysis identified the following as negative prognostic factors: histological grade, N classification, nodal status and treatment modalities. However, multivariate analysis determined positive nodal status, high histological grade and advanced N classification as the independent significant prognostic factors.

**Conclusion:**

Our results demonstrate several clinical and pathological features of PIOSCC and identify important prognostic factors associated with overall survival in PIOSCC. These prognostic factors include nodal status, histological grade, N classification, and treatment modalities, all of which are important for patient counseling and may be useful for the development of new treatment approaches.

## Introduction

Primary intraosseous squamous cell carcinoma (PIOSCC) is a rare type of odontogenic carcinoma arising from the jawbone, which is thought to develop from remnants of the odontogenic epithelium. This type of tumor was first described by Loos in 1913, and was renamed intraalveolar epidermoid carcinoma in 1948 by Willis[1]. The term primary intraosseous carcinoma was first recommended in 1972 by the World Health Organization (WHO)[2]. In 1989, the term intraosseous mucoepidermoid carcinoma was included as an additional type of primary intraosseous squamous cell carcinoma, by Waldron and Mustoe[3]. In the latest WHO classification published in 2005, PIOSCC replaced the old terms, and there are 3 subcategories. These include solid type tumors that invade the marrow spaces and induce bone resorption, squamous cell carcinoma (SCC) arising from the lining of an odontogenic cyst and in other odontogenic cysts, and SCCs in association with other benign epithelial odontogenic tumors[4]. A definitive diagnosis of PIOSCC can be difficult, as the lesion must be differentiated from alveolar carcinomas that have invaded the bone from the overlying soft tissue, tumors that have metastasized to the jaw from distant sites, tumors of the maxillary sinus, and other odontogenic tumors[5]. Due to the rarity of the disease, it is difficult to attain comprehensive understanding of the clinical and pathological features. Previous reports have described single case studies or studies with small sample sizes with the purpose to share the experience of management of PIOSCC, or attempt to depict different aspects of the disease, including clinical, histologic, radiologic, therapeutic, prognostic features[6–8], and some researchers conducted a retrospective review of the published reports to summarize the demographic features of PIOSCC[9].In the current report, we present a study investigating 77 patients with PIOSCC, to identify clinic-pathological features associated with adverse survival, especially in relation to pre-treatment risk factors, histologic features, TNM classification according to the American Joint Committee on Cancer (AJCC) classification system, tumor site, and treatment modalities.

## Materials and Methods

### Ethics Statement

Institutional review board approval was gained for this study from Nanjing Stomatological Hospital Ethics Committee. The data was analyzed anonymously, and therefore no additional informed consent was required.

### Materials

We identified 287 patients with mandible and maxilla malignancies who were treated at our treatment center between 2005 and 2015, and 77 patients diagnosed with PIOSCC were included in the study. Clinical, pathological, treatment modality and follow-up data were obtained from patient medical records. These included sex, presenting symptoms, age at diagnosis, TNM classification, histological grade, nodal status, use of adjuvant radiotherapy or chemotherapy, recurrence, and metastasis. Disease was re-classified by 2 experienced doctors using the TNM staging system according to the 7th Edition AJCC classification in 2010[10]. Two medical radiologists of plentiful experience gave an elaborate report on imaging characteristics including the extent and border of the tumor, the invaded adjacent tissue and so on. Histological grading of each tumor was evaluated and agreed on by 2 pathologists using the WHO grading criteria, with specific focus given to designating tumor grade and nodal status[11]. As for the high malignance of the disease, the follow-up work was conducted every 6 months until the expected outcome happened.

### Statistical analysis

The Kaplan–Meier product limit method was utilized to generate the overall survival curves. Statistical significance was distinguished by log-rank test. To evaluate the effect of prognostic factors on overall survival (OS), univariate and multivariate hazard ratios (HR) were obtained by a Cox regression model. On multivariate regression analysis, backward method (likelihood ratio) was used with p<0.05 removal from the model. Correlations between parameters and endpoints were assessed by the 2-tailed Fisher exact test. Statistical analysis was performed by the Statistical Product and Service Solutions software (SPSS Inc, Chicago, Illinois, USA). A p-value < 0.05 was considered statistically significant.

## Results

### Patient demographics

**Fig 1:**
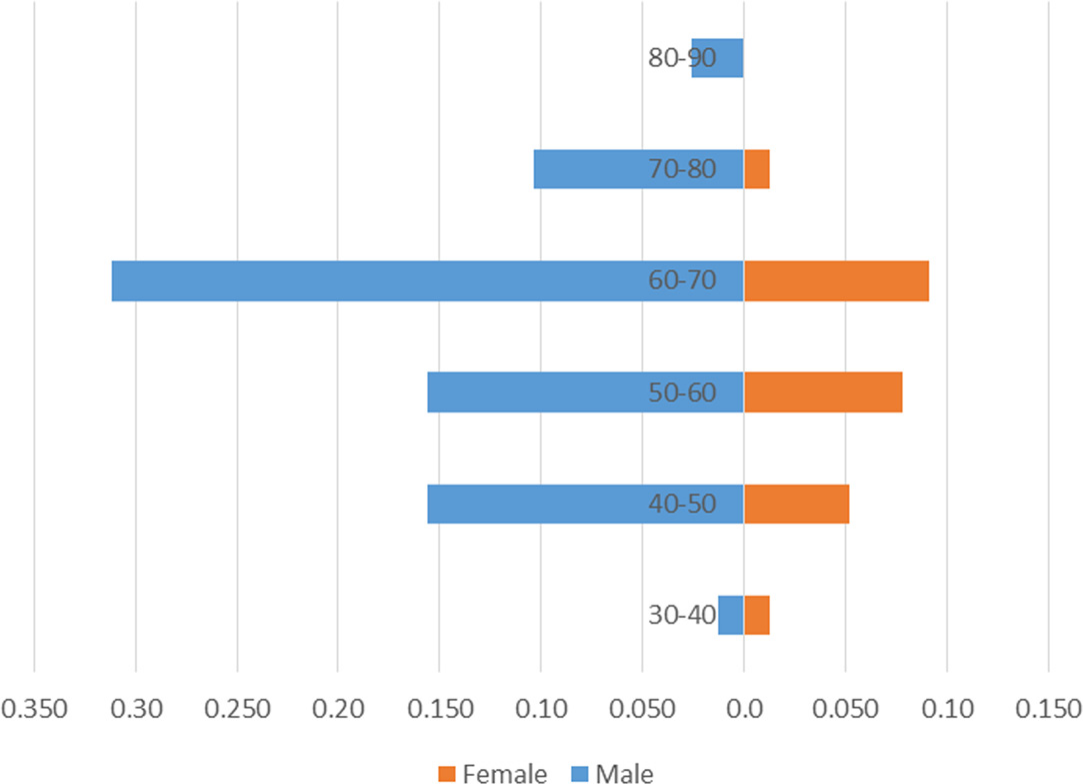
Age and sex distribution of the 77 patients with PIOSCC.

Clinical characteristics of patients are presented in **Table 1**. A total of 77 patients with PIOSCC were reviewed. The mean age at diagnosis was 58.8 years (range, 37−81 years). Of the 77 patients, 41 patients (53.2%) were ≥ 60 years and 58 patients (75.3%) were male. Males aged 60−70 years, were determined as the most susceptible members of population (**Fig 1**). Fifty-five tumors (71.4%) were located in the mandible, and 22 tumors (28.6%) occurred in the maxilla. Of the 55 mandibular tumors, 41 (74.5%) involved the posterior mandible, and 14 (25.5%) occurred in the anterior region. Of the 22 maxillary tumors, 10 (45.5%) occurred in the anterior area and 12 (54.5%) occurred in the posterior. The most common presenting symptom was jaw swelling (79.2%), followed by lip or facial numbness (48.1%). Some patients also complained of non-healing oral ulceration or extraction sockets at the time of diagnosis. All of the tumors were T4a lesions (moderately advanced local disease), which had invaded through the cortical bone of jaws, and the typical radiographic images of PIOSCC by computed tomography scan could be seen in **S1 Fig.** Seventy-four percent of patients had N0 stage disease, and N1 stage and N2 stage accounted for 10.4% and 15.6% of patients, respectively. The majority of patients (96.1%) showed no metastatic disease at the time of diagnosis.

**Table 1:** Clinical characteristics of PIOSCC.

Characteristics	No. (%) of patients
Age >60 years	
Yes	41 (53.2)
No	53 (46.8)
Sex	
Male	58 (75.3)
Female	19(24.7)
Tumor location	
Mandible	55 (71.4)
Maxilla	22 (28.6)
Smoking	
Yes	19 (24.7)
No	58 (75.3)
Jaw swelling	
Yes	61 (79.2)
No	16 (20.8)
Lip or facial numbness	
Yes	37 (48.1)
No	40 (51.9)
Oral ulceration	
Yes	23 (29.9)
No	54(70.1)
T classification	
T4a	77(100)
N classification	
N0	57 (74.0)
N1	8 (10.4)
N2	12 (15.6)
M classification	
M0	74 (96.1)
M1	3 (3.9)
NR not reported	

### Treatments

Pathological and treatment data are presented in **Table 2**. All the patients underwent surgery at the tumor site, with 46.8% undergoing partial or total maxillectomy or mandibulectomy, and 53.2% of patients undergoing segmental resection. All patients underwent a neck dissection, of which 66.2% were selective neck dissections and 33.8% were radical neck dissections. In patients with N 1/2 stage, radical neck dissection was essential in all cases, while 89.5% of patients with N 0 stage disease underwent selective neck dissection. Forty-three patients (55.8%) were treated by surgery alone. Nineteen patients (24.7%) received radiotherapy only, and 6 patients received chemotherapy as postoperative adjuvant treatment. Nine patients (11.7%) received both chemotherapy and radiotherapy after surgery. In the present series, for patients who received radiotherapy, the mean dose was 64Gy by using Intensity Modulated Radiation Therapy(IMRT). The main chemotherapy agent was cisplatin plus paclitaxel. Briefly, paclitaxel was used 150mg/m^2^ on Monday and cisplatin 70mg/m^2^ on Tuesday, patients receive that treatment for 6 consecutive weeks. Histological grade was one of the most important factors for determining a post-surgical treatment plan. Histological grade 3 required adjuvant radiotherapy in 80% of cases, and only 45.2% and 24.4% of patients with grade 2 and grade 1 disease, respectively, underwent similar therapy. Similarly, adjunct chemotherapy use was decreased in grades 1 and 2, compared to grade 3 tumors (12.2% and 22.6%, versus 60%, respectively).

**Table 2:** Pathologic and treatment characteristics of PIOSCC.

Characteristic	No. (%) of patients
Histopathology	
Grade 1	41 (53.2)
Grade 2	31 (40.3)
Grade 3	5 (6.5)
Nodal status	
Positive	54 (70.1)
Negative	23 (29.9)
Surgery	
Maxillectomy or mandibulectomy	36 (46.8)
Segmental resection	41 (53.2)
Radical neck dissection	26 (33.8)
Selective neck dissection	51 (66.2)
Radiotherapy	
Yes	28 (36.4)
No	49 (63.6%)
Chemotherapy	
Yes	62 (80.5)
No	15 (19.5)
Treatment	
Surgery alone	43 (55.8)
Surgery + Radiotherapy	19 (24.8)
Surgery + Chemotherapy	6 (7.8)
Surgery + Radiotherapy + Chemotherapy	9 (11.7)
Recurrence	
Yes	10 (13.0)
No	67 (87.0%)
Last contact	
No evidence of disease	26 (33.8)
Living with disease	16 (20.8)
Died of disease	33 (42.9)
Other	2 (3.5)
NR not reported	

### Outcomes

**Fig 2:**
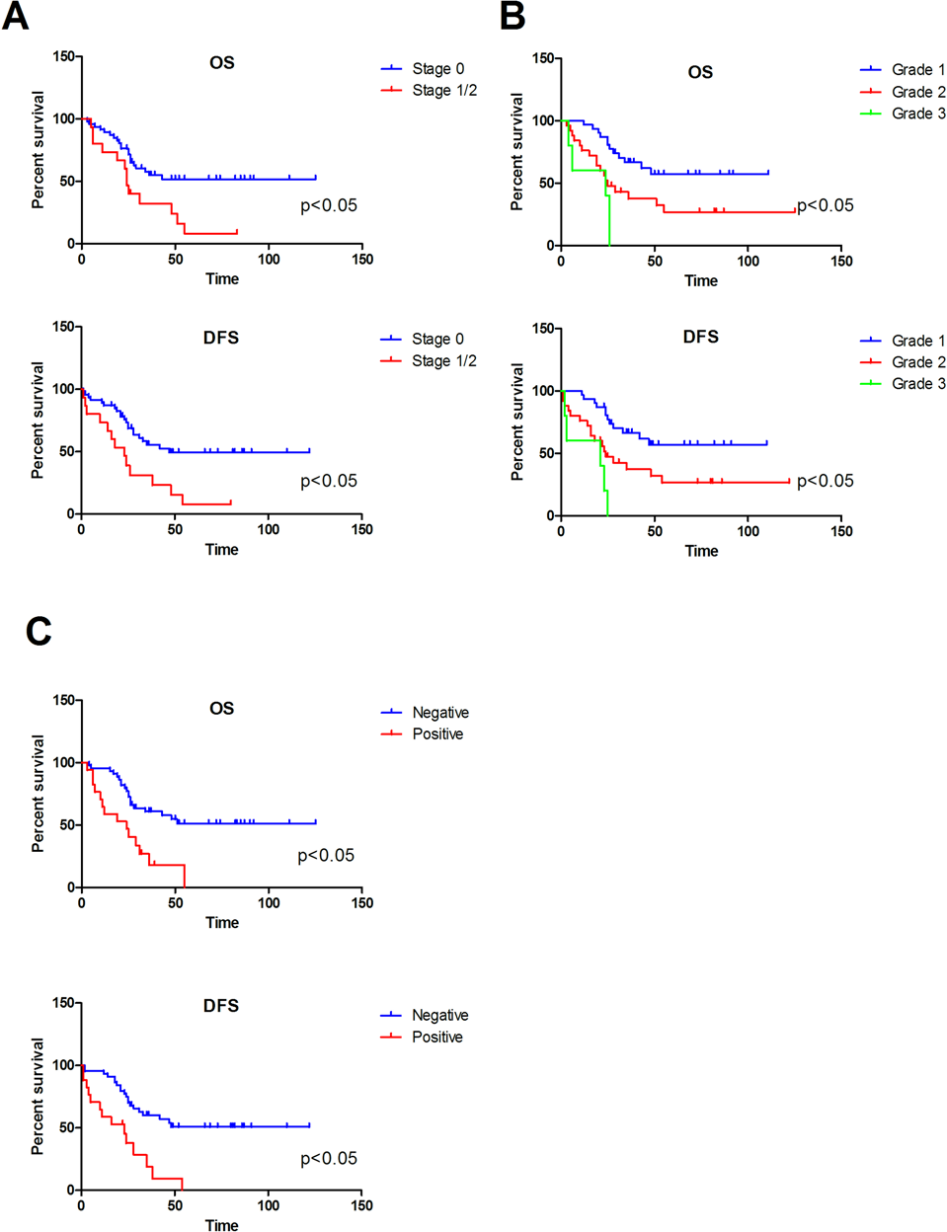
Overall and disease-free survival by (A) N classification, (B) histological grade and (C) nodal status.

Outcome data are presented in **Tables 3 and 4**. When identifying the clinic-pathologic features of PIOSCC, all 77 patients were included, but when determining the prognostic factors of PIOSCC, 16 patients were excluded, among which 6 patients were lost to follow up at the very beginning of follow-up work, 7 patients diagnosed with PIOSCC in 2015 and 3 M1 stage patients. The median length of follow-up of 61 patients was 39.1 months. The estimated 2-year and 5-year overall survival were 68.9% and 38.8%, respectively. On univariate analysis, N classification, histological grade, nodal status and treatment modalities were significant prognostic factors for survival. Advanced N classification (HR 1.763[1.165–2.669], p = 0.007), high histological grading (HR 2.474 [1.491–4.105], p < 0.001), and positive nodal status (HR 3.247 [1.612–6.543], p = 0.001) decreased 5-year OS significantly (**Fig 2**). Similarly, all these factors significant decreased 5-year DFS. Treatment modalities also demonstrated a significant relationship with 5-year OS (HR 1.674 [1.260–2.224], p <0.001) and 5 year DFS (HR 1.666 [1.257–2.207], p <0.001). Patients who underwent surgery alone appeared to have a better survival rate than patients who received adjuvant radiotherapy and/or chemotherapy. The mortality rated noted during the period of adjuvant therapy was 8%.

**Table 3:** Survival analysis of clinical and histopathologic variables for PIOSCC.

Variable	OS (%)	p	HR (95% CI)	DFS (%)	p	HR (95% CI)
Sex						
Male	43.7			43.6		
Female	28.6	0.842	0.925 (0.432−1.984)	25.1	0.791	0.902 (0.421−1.934)
Tumor location						
Mandible	41.9			41.2		
Maxilla	32.9	0.835	0.928 (0.458–1.878)	32.9	0.853	0.935 (0.462–1.893)
Jaw swelling						
Yes	37.4			37.7		
No	45.7	0.759	1.139 (0.495−2.622)	42.9	0.781	1.126 (0.489−2.589)
Chronic inflammation						
Yes	54.3			54.4		
No	26.6	0.187	0.621 (0.306−1.260)	25.7	0.189	0.622 (0.307−1.264)
N classification						
0	48.9			48.9		
1/2	0	0.007	1.763 (1.165−2.669)	0	0.007	1.772 (1.171−2.683)
Histological Grade						
1	57.1			57.0		
2	26.8			26.5		
3	0	<0.001	2.474 (1.491−4.105)	0	<0.001	2.584 (1.542−4.329)
Nodal status						
Positive	0			0		
Negative	51.1	0.001	3.247 (1.612−6.543)	51.1	0.001	3.220 (1.599−6.482)
Treatment						
Surgery alone	64.6			64.6		
Surgery + R	0			0		
Surgery + C	0			16.7		
Surgery + R + C	0	<0.001	1.674 (1.260−2.224)	0	<0.001	1.666 (1.257−2.207)

Abbreviations: HR, hazards ratio; CI, confidence interval; R, radiotherapy; C, chemotherapy.

**Table 4:** Multivariate survival analysis of clinical and histopathologic variables for PIOSCC.

Variable	OS		DFS	
	p	HR (95% CI)	P	HR (95% CI)
N classification	0.012	0.243 (0.081−0.731)	0.016	0.256 (0.085−0.773)
Histological grade	0.006	2.193 (1.256−3.820)	0.004	2.275 (1.292−4.006)
Nodal status	0.001	18.172 (3.484−94.784)	0.001	16.209 (3.108−84.523)

Abbreviations: HR, hazards ratio; CI, confidence interval.

By contrast, no statistically significant difference was observed in 5-year OS or DFS between patients with and without the following clinical characteristics: sex, tumor location and jaw swelling. On multivariate analysis, positive nodal status (HR 18.172 [3.484–94.784], p = 0.001 for OS, HR 16.209[3.108–4.523] for DFS), high histological grade (HR 2.193 [1.256–3.820], p = 0.006 for OS, HR 2.275 [1.292−4.006] for DFS) and advanced N classification ((HR 0.243 [0.081–0.731], p = 0.012 for OS, HR 0.256 [0.085–0.773] for DFS) were found to be significantly poor prognostic factors for 5-year OS and DFS. Notably, among the three negative prognostic factors of PIOSCC, positive nodal status appeared to have a more profound impact on survival than histological grade and N classification for its high hazard ratio value.

## Discussion

PIOSCC is a rare malignant tumor arising from either of the jaws, and there is not any initial connection with the oral mucosa because the disease develops from remnants of odontogenic epithelium[9]. The aim of this study was to identify clinic-pathological factors for poor prognosis of PIOSCC. A definite diagnosis of PIOSCC is often challenging, as it is difficult to differentiate PIOSCCs from SCCs of surface mucosal origin and to rule out the possibility of other odontogenic carcinomas[12]. In general, the prognosis for PIOSCC is poor[13]. The estimated 2-year and 5-year survival rates of our study are similar to results published in other series with OS figures. According to Shear’s study in 1969, the 5-year survival was 30−40%[12]. Huang et al. reported 39 cases of PIOSCC and found that the overall survival rates of PIOSCC were 69.8% at 2 years and 36.3% at 5 years[14]. Bodner in 2011 retrieved and reviewed 96 articles d escribing 116 cases and their finding showed that the overall survival rate was 62% at 2 years and 38% at 5 years[9]. However, most authors just focused on the 5-year survival rate and paid little attention to potential prognostic factors of the disease, and some authors lacked in the numbers of the patients of PIOSCC, making their study not so convinced and precise. For the reasons above, we determined to study larger number of the disease and contribute to the work. On univariate analysis, our results identified several clinical and pathologic factors associated with decreased survival including advanced N classification, high histological grade, positive nodal status and treatment modalities. However, multivariate analysis showed positive nodal status, histological grade and advanced N classification were found to be the independent prognostic factors for survival rate. The results of survival analysis were partially similar to those studies in which histological grading and lymph node metastasis were suggested as prognostic indicators[13, 14]. However, there are also some limitations of our study must be mentioned. As mentioned in Woolgar’s review, PIOSCC has three subcategories: PIOSCC ex odontogenic, PIOSCC ex keratocystic odontogenic tumor, solid type. In fact, different types of PIOSCC have different clinic-pathological features thus having different prognosis[15]. Our study gave specific focus to histological grading, designating tumor grade and nodal status but did not identify the subtypes of the disease, so further studies needs to be conducted. Another potential bias that exists in our study was that the follow up time was not long enough and the sample size needed to be increased.

In the present study, demographic factors were also evaluated for effect on prognosis, but did not appear to show significant relationships with survival. Clinical features including jaw swelling, non-healing ulceration and tumor location were also analyzed, but no significant relationship with survival rate was observed. Further studies are needed to identify potential confounders.

In the treatment modalities, patients who received adjuvant radiotherapy or chemotherapy showed a trend toward worse OS than those who received surgery only. This may be due to patients receiving adjuvant radiotherapy or chemotherapy having tumors with more aggressive features, high-risk pathologies or advanced disease stage. In terms of the chemotherapy agents, at present, the three-drug regimen consisting of a taxel (including docetaxel), cisplatin and 5-fluorouracil (TPF) is recommended in head and neck squamous cell carcinoma(HNSCC) chemotherapy, and many clinical trials in 2006 or 2007 have verified that the TPF regimen improved the outcome of patients with HNSCC[16–18]. Before that, chemotherapy with cisplatin plus 5-fluorouracil(PF) was widely accepted as the effective therapy for HNSCC[19–21]. In the present series, we chose the cisplatin and paclitaxel as the main chemotherapy agents, which was proved as effective as the classical PF regimens by several clinical trials[22, 23].In general, surgery remains as the requisite form of treatment and patients should be treated in accordance with the protocol for advanced head and neck tumor, although further studies are necessary for the further development of treatment modalities.

## Conclusion

Primary intraosseous squamous cell carcinoma is a rare malignant tumor. The estimated 2-year and 5-year overall survival were 68.9% and 38.8%, respectively. Positive nodal status, high histological grade and advanced N classification all portend poor prognosis among patients with PIOSCC. Taking these factors into consideration, we can be more expert in patient counseling on disease prognosis and improving targeted aggressive therapy. The influence of postoperative adjuvant radiotherapy or chemotherapy remains uncertain, and further studies are needed for the optimization of treatment strategies.

## Supporting Information

S1 Fig Typical radiographic images of PIOSCC by Computed Tomography Scan. PIOSCC in the left retromolar region of the mandible. (a)Axial CT section, (b) Sagittal CT section, (c) Coronal CT section (d)3D reconstructed CT image.
(TIF)

S1 File Data. (XLSX)

S2 File STROBE checklist. (DOC)

S3 File Ethics Approval Document. (DOCX)
