# Supplementary figures and images for: Prognostic Factors of Primary Intraosseous Squamous Cell Carcinoma (PIOSCC): A Retrospective Review
Source: PLoS One. 2016 Apr 13;11(4):e0153646. doi: 10.1371/journal.pone.0153646 (PMC4830592; doi:10.1371/journal.pone.0153646)

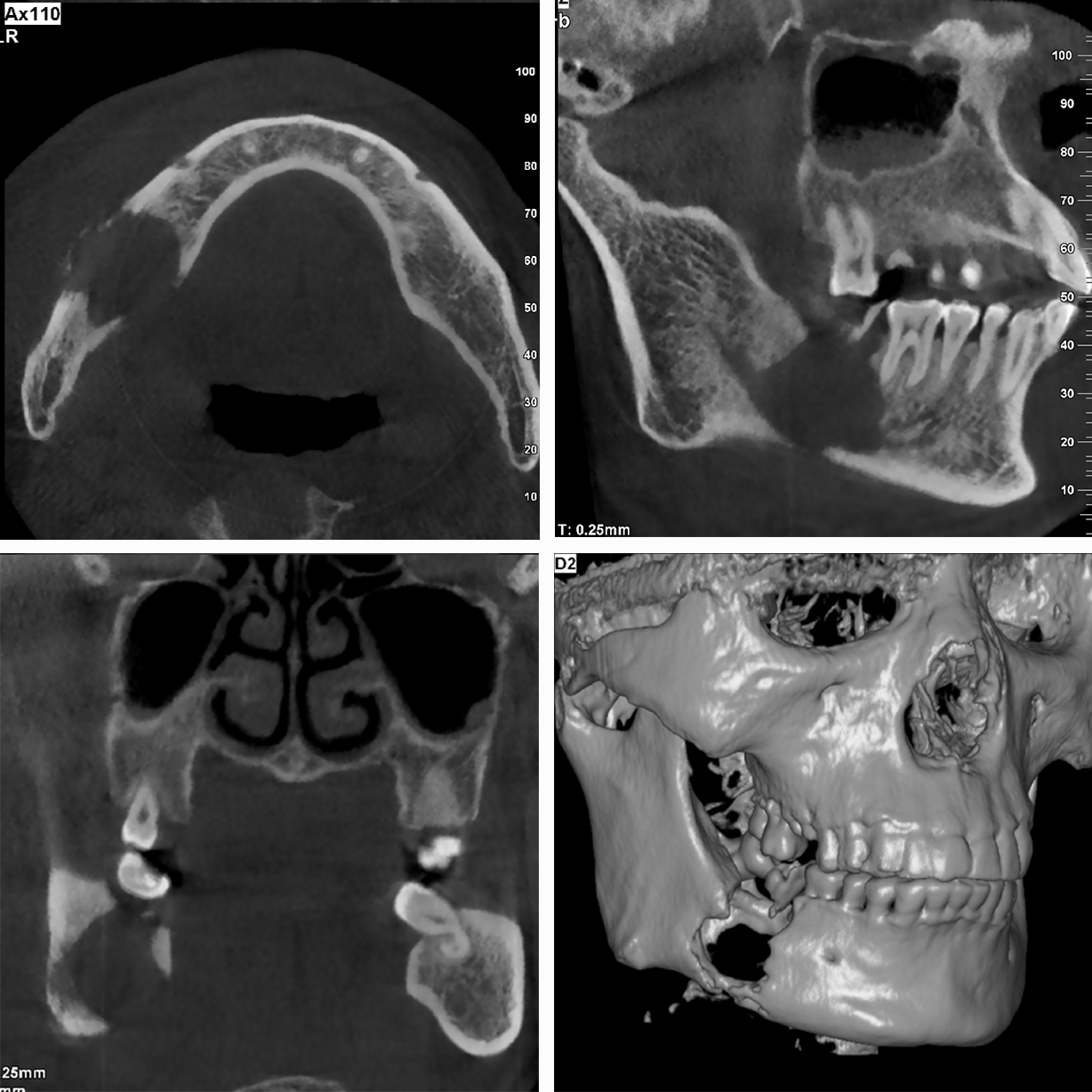

Supplement: S1 Fig — PIOSCC in the left retromolar region of the mandible. (a)Axial CT section, (b) Sagittal CT section, (c) Coronal CT section (d)3D reconstructed CT image. (TIF) [file pone.0153646.s001.tif]
