# Supplementary material for: Prognostic Factors of Primary Intraosseous Squamous Cell Carcinoma (PIOSCC): A Retrospective Review
Source: PLoS One. 2016 Apr 13;11(4):e0153646. doi: 10.1371/journal.pone.0153646 (PMC4830592; doi:10.1371/journal.pone.0153646)
Supplement: S3 File — (DOCX) [file pone.0153646.s004.docx]

Nanjing Stomatological Hospital Ethics Committee Approval

Approval Number: 2015NL-003KS

| Project name | A study on the prognostic factors of primary intraosseous squamous cell carcinoma (PIOSCC). | | |
| --- | --- | --- | --- |
| Host Department | Oral Maxillofacial Surgery | Project Leader | Wei Han |
| Materials | 1.Submission letter (including submission document list, main researcher signature and sender signature)  2. Application form  3. Clinical research plan  4. Informed consent  5. Case report form  6. Materials to study members  7. Main researchers’ resume (dated signature and Good Clinical Practice (GCP) certificate)  8. Criteria of assessment  9. Animal experiment reports  10. Investigator's Brochure(review of preliminary work) | | |
| Opinions of Nanjing Stomatological Hospital Ethics Committee | Voter turnout: 2 Approval: 2 Disapproval: 0 Abstention: 0 | | |
|  | Review Method：(1)Meeting (2) Quick Code Review(√)  Review Result : (1) Approval （√） (2) Approval after some amendments (3)Disapproval (4) Terminate or suspend the approved trial.  Nanjing Stomatological Hospital Ethics Committee  2015-03-13 | | |
|  | Note：Audit trail reports need to be submitted from the experiment day according to GCP’s rules. | | |
